# Prevalence and Severity of Potential Drug–Drug Interactions in Patients with Multiple Sclerosis with and without Polypharmacy

**DOI:** 10.3390/pharmaceutics14030592

**Published:** 2022-03-08

**Authors:** Paula Bachmann, Niklas Frahm, Jane Louisa Debus, Pegah Mashhadiakbar, Silvan Elias Langhorst, Barbara Streckenbach, Julia Baldt, Felicita Heidler, Michael Hecker, Uwe Klaus Zettl

**Affiliations:** 1 Section of Neuroimmunology, Department of Neurology, Rostock University Medical Center, Gehlsheimer Str. 20, 18147 Rostock, Germany; niklas-frahm@gmx.de (N.F.); janedebus@freenet.de (J.L.D.); pegah.mashhadiakbar@uni-rostock.de (P.M.); silvan.langhorst@uni-rostock.de (S.E.L.); babswehr@web.de (B.S.); julia.baldt@outlook.de (J.B.); michael.hecker@rocketmail.com (M.H.); uwe.zettl@med.uni-rostock.de (U.K.Z.); 2 Ecumenic Hainich Hospital Mühlhausen, Pfafferode 102, 99974 Mühlhausen, Germany; f.heidler@oehk.de

**Keywords:** multiple sclerosis, polypharmacy, drug–drug interactions, clinical decision support software, over-the-counter drugs, Rx drugs

## Abstract

Polypharmacy (PP) is a common problem in modern medicine, especially known to affect patients with chronic diseases such as multiple sclerosis (MS). With an increasing number of drugs taken, the risk of potential drug–drug interactions (pDDIs) is rising. This study aims to assess the prevalence and clinical relevance of polypharmacy and pDDIs in patients with MS. Pharmacological data of 627 patients with MS were entered into two drug–drug-interaction databases to determine the number and severity of pDDIs for each patient. The patients were divided into those with and without PP (total PP and prescription medication PP (Rx PP)). Of the 627 patients included, 53.3% and 38.6% had total PP and Rx PP, respectively. On average, every patient took 5.3 drugs. Of all patients, 63.8% had at least one pDDI with a mean of 4.6 pDDIs per patient. Less than 4% of all pDDIs were moderately severe or severe. Medication schedules should be checked for inappropriate medication and for possible interacting drugs to prevent pDDIs. Physicians as well as pharmacists should be more sensitive towards the relevance of pDDIs and know how they can be detected and avoided.

## 1. Introduction

Polypharmacy (PP) is the simultaneous use of multiple drugs and mostly defined as the intake of at least five drugs at the same time [1]. PP particularly affects elderly people with various illnesses and comorbidities and those with severe chronic diseases, such as patients with multiple sclerosis (MS) [2,3]. Within recent years, life expectancy has continued to rise and the prevalence of MS has increased [4]. Therefore, the significance of PP in elderly and patients with MS is also increasing. Globally, there are approximately 2.8 million patients diagnosed with MS [4], which is an immune-mediated disease affecting the central nervous system, causing demyelination, oligodendrocyte loss, synaptic and axon loss, as well as reactive gliosis [5]. As these processes are neither limited in time nor in location, symptoms of MS vary a lot [6] and the disease progression can hardly be predicted [7,8,9]. MS leads to an accumulation of disability, either with or without the occurrence of relapses. The disease course can be distinguished into three main subtypes [10]: the relapsing–remitting course (RRMS), the primary progressive course (PPMS) and the secondary progressive course (SPMS). An initial clinical episode with symptoms suggestive of MS is referred to as clinically isolated syndrome (CIS) [11]. 

There is still no cure for MS, but there are treatments that can alleviate the symptoms, prevent relapses and delay disease progression. Disease-modifying drugs (DMDs) for MS are immunosuppressive or immunomodulatory [12,13,14]. While there are several DMDs available to treat patients with RRMS and SPMS [15,16], there is currently only one drug approved for patients with PPMS (ocrelizumab) [17]. Aside from DMDs, MS patients mostly require symptomatic drugs as well as medication for comorbidities. Patients with late-stage MS often suffer from multiple symptoms, such as gastrointestinal, psychiatric and motoric complaints [18]. The use of several symptomatic drugs in combination with DMDs and comorbidity therapeutics can quickly lead to PP in patients with MS [6,19]. Previous studies have shown that PP rates in MS patients range from 15% to 59% [2]. With an increasing number of drugs taken and with PP, the risk of potential drug–drug interactions (pDDIs) is increasing as well [19]. 

pDDIs are possible interactions between two or more drugs taken in combination. Both pharmacodynamic and pharmacokinetic interactions are possible between different drugs. This can lead to a change in the effectiveness of the drugs used [20], which makes pDDIs potentially dangerous. Drug effects can either be attenuated or potentiated, increasing the risk of treatment failure and side effects, respectively [21]. pDDIs are a significant but frequently underestimated risk factor for hospitalizations and secondary comorbidities [22,23]. It is estimated that between one and two percent of hospitalizations are caused by pDDIs [24]. To date, there is very limited information on the relevance of pDDIs in patients with MS. To our knowledge, there are no studies on pDDIs in patients with MS that were conducted in a large cohort.

The objective of our study was to investigate the prevalence of pDDIs as well as the degrees of pDDI severity in patients with MS. We compared patients with PP (PwP) and patients without PP (Pw/oP). PP status was evaluated from two different perspectives: one including only prescription drugs and one that also included over-the-counter (OTC) drugs. We aimed to find out the most frequently used drugs and the most often-occurring pDDIs. In addition, we provide estimates of the risks of PwP versus Pw/oP for having more severe pDDIs. 

## 2. Materials and Methods

### 2.1. Study Population

This cross-sectional multicenter study was conducted at the Department of Neurology of the University Medical Center Rostock (Germany) and at the Department of Neurology of the Ecumenic Hainich Hospital Mühlhausen (Germany). In total, data of 627 patients with MS were collected in both medical centers from March 2017 to May 2020. Inclusion criteria were an age of at least 18 years and a diagnosis of CIS or MS according to the revised McDonald criteria [11]. Both inpatients and outpatients were asked to participate in this study. Data acquisition was performed during clinical routine appointments in case of outpatients and during a clinical stay (due to routine glucocorticosteroid pulse therapy, a recent relapse or progression of disability) in case of inpatients. 

The study was approved by the ethics committees of the University of Rostock and of the Physicians’ Chamber of Thuringia (permit numbers A 2014-0089 and A 2019-0048). Furthermore, our study was conducted according to the Declaration of Helsinki. All patients participated voluntarily and provided informed consent.

### 2.2. Gathered Data

We collected sociodemographic, clinical and pharmaceutical data of the 627 patients by assessing each patient’s medical record and by conducting a clinical examination as well as a structured interview.

Sociodemographic data consisted of age, sex and partnership. Moreover, number of children and siblings, employment status, educational level and number of school years, as well as place of residence (divided into rural community, provincial town, medium-sized town or city) were recorded. Clinical data contained comorbidities, disease duration, age at MS onset, disease course (CIS, PPMS, RRMS or SPMS) and clinical disability according to Kurtzke’s Expanded Disability Status Scale (EDSS) [25]. Comorbidities were ascertained according to the definition by Moss et al. [26,27] through patient interviews and patient records and summarized into 16 comorbidity groups based on the affected organ system. Pharmaceutical data comprised each patient’s medication schedule, including drug names and active agents, dosage and application form as well as the indication for every drug. Drugs were classified according to the anatomical therapeutic chemical (ATC) classification system. Drugs that were not only available on prescription as well as nutritional supplements were also included.

### 2.3. Drug Characterization

For drug classification, we collected additional data on all drugs to arrange them by the following criteria: OTC drugs and prescription-only drugs (Rx). Besides the indication, we also noted the treatment goal for each drug: DMDs, symptomatic relief, or treatment of comorbidities or other conditions (e.g., contraception). Drugs were also classified according to the intake interval. Long-term drugs are those permanently taken, either daily or in routine intervals, e.g., weekly or every three months, while drugs on demand (*pro re nata*, PRN) are those taken when needed to care for acute symptoms.

### 2.4. Polypharmacy

In our study, we differentiated between total PP and Rx PP. As it is the most common definition in the literature [1], we defined total PP as the simultaneous intake of at least five drugs (Rx and OTC drugs considered together). Rx PP was defined as the intake of at least five drugs prescribed by a doctor (neglecting OTC drugs that were not prescribed by a doctor).

### 2.5. Identification of Drug–Drug Interactions

To identify pDDIs, we used two different kinds of software: a clinical decision support software (CDSS) called *MediQ* and a drug–drug interaction database (DDID) called *Stockley’s Interaction Checker*. Each patient’s medication schedule was checked with *MediQ* and *Stockley’s*. *MediQ* is a Swiss web-based interaction checker for drug–drug, drug–food, drug–alcohol and drug–polymorphism interactions. It includes more than 2000 active substances and more than 49,000 interactions [28]. The following pDDI categories are distinguished in *MediQ*: high danger, average danger, low danger, no danger of interaction and lack of evidence. *MediQ* is one of the most commonly used CDSS in the German-speaking area and has found to be the most complete in a study comparing five CDSS in German language [29]. To achieve greater coverage, we also used *Stockley’s Interaction Checker* by the Royal Pharmaceutical Society, a British online tool based on the comprehensive and evidence-based compendium *Stockley’s Drug Interactions*. This online tool contains information on drug–drug, drug–herb, drug–alcohol and drug–food interactions, with about 85,000 interactions listed in total [30]. In *Stockley’s Interaction Checker*, there are the following severity rating categories: severe, moderate, mild and no interaction. As both databases are permanently updated, it is important to note that we checked the medication plans of our patients for pDDIs between May and November 2020.

### 2.6. Composite Rating of pDDI Severity Levels

To combine each pDDI’s severity level from *MediQ* and *Stockley’s Interaction Checker*, we allocated each severity rating to a numeric value: 3 for severe (*Stockley’s*) or high danger (*MediQ*); 2 for moderate (*Stockley’s*) or average danger (*MediQ*); 1 for mild (*Stockley’s*) or low danger (*MediQ*); and 0 for no danger of interaction or lack of evidence. Then, for the combined severity grade, the numeric values of the severity levels of both databases were summed for each pDDI. A sum of ≤2, 3, 4, 5 and 6 indicated a mild, mildly moderate, moderate, moderately severe and severe pDDI, respectively.

### 2.7. Statistics

Using SPSS Statistics version 27 (IBM) and R version 3.6 (R Foundation for Statistical Computing), the pseudonymized data were statistically analyzed. Counts and percentages were calculated for descriptive purposes. Measures of location and dispersion such as median, range, mean value and standard deviation were calculated as appropriate. Frequencies were also calculated to assess the prevalence of drugs used, pDDIs and pDDI severity levels. For comparing PwP and Pw/oP, statistical testing was applied. For numerical variables, we applied two-sample two-tailed Welch *t*-tests and Mann–Whitney U tests. For categorical variables, we used Fisher’s exact tests and chi-squared tests. The significance level was set at α = 0.05. The analyses should be considered as exploratory. Bar charts and pie charts were created with Microsoft Excel version 16.49. Confidence intervals for proportions were calculated according to the Clopper–Pearson exact method and visualized using R version 3.6.

## 3. Results

### 3.1. Sociodemographic and Clinical Patient Profile

Of all 627 patients included, 70.3% were female and 29.7% were male (Table 1). The patients’ ages ranged from 19 to 86 years, with a mean of 48.6 years (standard deviation: 13.3 years). Most patients lived in a partnership (74.2%) and in a rural community (35.7%). Almost half of all patients had one sibling (48.6%) and at least two children (45.9%). On average, the patients went to school for 10.5 years. A subset of 63.5% of all patients were trained as a skilled worker. About half of all patients (48.5%) obtained disability pension at the time of the data acquisition. Among the patients analyzed, the median EDSS score was 3.5 (range 0 to 9.0) and the median disease duration was 10 years, with a median age of 35 years at disease onset. Most patients (66.2%) were diagnosed with a CIS (N = 27) or RRMS (N = 388). The number of comorbidities varied from zero to nine, with a median of one comorbidity besides MS.

### 3.2. Polypharmacy

By the definition of total PP, a slight majority of 53.3% of all patients had PP. According to our definition of Rx PP, 38.6% of the patients had PP, leaving 61.4% of Pw/oP. The sex ratios in all four groups (total PP, no total PP, Rx PP, no Rx PP) were nearly the same as in the total population. However, we found that PwP were, on average, significantly older than Pw/oP (*t*-test: *p* < 0.001) for total PP as well as for Rx PP (53.0 years vs. 43.6 years and 54.8 years vs. 44.7 years, respectively). PP was also associated with a significantly lower number of years in school (*t*-test: *p* < 0.001) and a lower educational level (chi-squared test: *p* < 0.02). Moreover, PwP were more than twice as likely to receive disability pension compared to Pw/oP (67.4% vs. 27.0% for total PP and 73.6% vs. 32.7% for Rx PP, respectively) and were employed more rarely (chi-squared test: *p* < 0.001). In PwP, the median EDSS score was significantly higher (4.5 vs. 2.0 for total PP; 5.0 vs. 2.5 for Rx PP, Mann–Whitney U test: *p* < 0.001 for both) and the median disease duration was longer (12.5 vs. 9 years for total PP; 14 vs. 9 years for Rx PP, Mann–Whitney U test: *p* < 0.001), indicating that PwP were typically at an advanced stage of MS than Pw/oP. The median number of comorbidities was also significantly higher in PwP in comparison to Pw/oP (Table 1).

### 3.3. Comorbidities

With a prevalence of 27.1%, cardiovascular comorbidities were most frequent among all patients, followed by psychiatric (19.3%), metabolic (17.7%), neurological and orthopedic comorbidities (both 12.4%; Supplementary Table S1). PwP (both total PP and Rx PP) were significantly more likely to have one of these aforementioned comorbidities than Pw/oP (Fisher’s exact test: *p* ≤ 0.027). Moreover, gastrointestinal and ophthalmological comorbidities were also significantly more prevalent among PwP. PwP and Pw/oP had very different distributions of the numbers of comorbidities. For instance, the proportion of patients without any comorbidities was 3 to 4 times higher for Pw/oP than for PwP (Table 1).

### 3.4. Drug Profile

The number of drugs taken per patient ranged from 0 to 19, with a mean of 5.3 drugs per patient (Table 1). A subset of 46.7% of the patients took zero to four drugs (no total PP) and 53.3% took at least five drugs (total PP). More than nine drugs were taken by 11.6% of all patients, resulting in so-called excessive PP.

In total, the 627 patients used 3341 drugs, counted with repetitions (Table 2). Of all drugs, 85.5% were long-term drugs and 14.5% were PRN drugs. As many as 78.7% of all drugs taken were on prescription. Those drugs were significantly more often used by PwP (both total PP and Rx PP), while OTC drugs accounted for a higher proportion in Pw/oP as compared to PwP (24.7% vs. 20.3% and Fisher’s exact test: *p* = 0.011 for total PP, 32.6% vs. 14.3% and Fisher’s exact test: *p* < 0.001 for Rx PP). A subset of 46.6% of all drugs were used to treat comorbidities. About 37.5% of all drugs were symptomatic drugs and 15.9% were DMDs. Symptomatic and comorbidity drugs were more frequently used by PwP than by Pw/oP (symptomatic drugs: 39.9% vs. 29.0% for total PP, comorbidity drugs: 48.6% vs. 39.9% for total PP, respectively).

The most often used non-DMD was cholecalciferol, which was taken by 41.6% of all patients, followed by pantoprazole (28.4%) and enoxaparin (20.3%). These medications were used significantly more often by PwP than by Pw/oP (Table 3). A total of 123 patients (19.6%) received methylprednisolone. A frequently used DMD was interferon beta-1a, which was predominantly applied by Pw/oP (13.3% in Pw/oP vs. 7.5% in PwP for total PP, Fisher’s exact test: *p* = 0.018; 13.0% in Pw/oP vs. 5.8% in PwP for Rx PP, Fisher’s exact test: *p* = 0.004). The DMD glatiramer acetate was also taken significantly more frequently by Pw/oP than by PwP (Fisher’s exact test: *p* = 0.012 for total PP, *p* = 0.023 for Rx PP) (Table 3).

Most patients used drugs for disorders related to alimentary tract and metabolism (e.g., proton pump inhibitors or antidiabetic drugs); 68.9% of all patients took at least one drug from this group. The second most commonly taken medication group comprised antineoplastic and immunomodulating agents (62.2%), to which interferons and mitoxantrone belong, followed by drugs affecting the nervous system (54.1%). The latter included, for example, analgesics such as acetylsalicylic acid or acetaminophen, and antidepressants such as citalopram.

### 3.5. Drug–Drug Interactions

**Figure 1:**
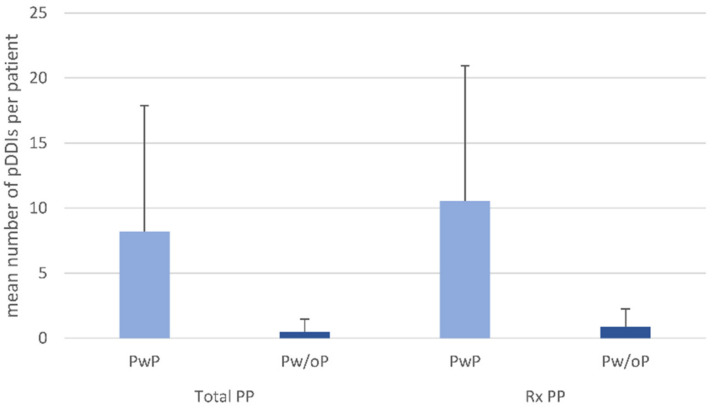
Average number of pDDIs per patient with MS, stratified by PP status and type of PP. The patients were classified by PP status according to total PP (intake of at least five drugs of any kind) and Rx PP (intake of at least five drugs only available on prescription). Standard deviations are displayed as error bars. The average number of pDDIs was higher in PwP compared with Pw/oP, for both total PP (8.2 ± 9.7 versus 0.5 ± 1.0) and Rx PP (10.5 ± 10.4 versus 0.9 ± 1.4). MS—multiple sclerosis; pDDI—potential drug–drug interaction; PP—polypharmacy; PwP—patients with polypharmacy; Pw/oP—patients without polypharmacy; Rx—only available on prescription.

For the 627 patients analyzed, we detected a total of 2887 pDDIs (counted with repetitions). These resulted from 1424 different pDDIs (counted without repetitions) that were related to 267 different active agents (Supplementary Table S3). For 63.8% of all patients, at least one pDDI was identified. The number of pDDIs per patient ranged from 0 to 65, with a mean value of 4.6 pDDIs per patient. Of all 2887 recorded pDDIs, 2745 (95.1%) applied to patients with total PP, while they only made up around half of all patients (53.3%). With regard to Rx PP, the PwP group (38.6% of all patients) accounted for 2550 (88.3%) of all pDDIs. Accordingly, the average number of pDDIs differed considerably between PwP and Pw/oP (Rx PP as well as total PP). In Pw/oP, the average number of pDDIs was 0.5 ± 1.0 (total PP) and 0.9 ± 1.4 (Rx PP) per patient; in PwP, it was 8.2 ± 10.7 (total PP) and 10.5 ± 10.4 (Rx PP) (Figure 1).

**Figure 2:**
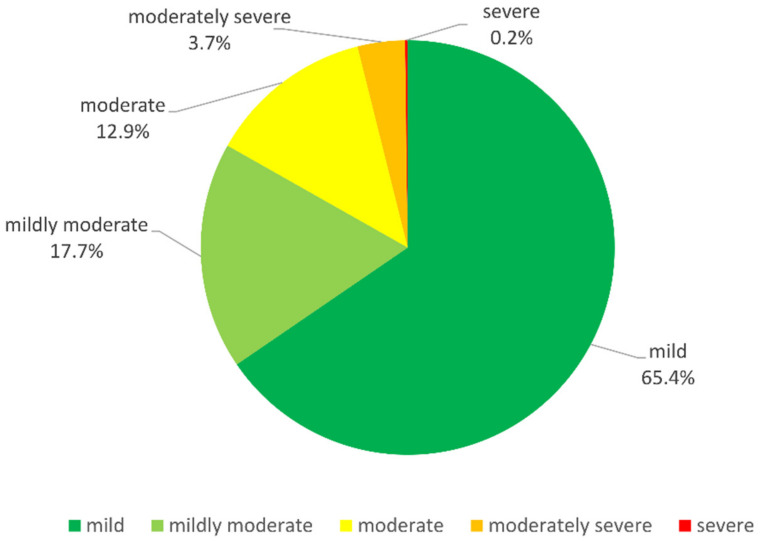
Distribution of severity levels of pDDIs in patients with MS. For the 627 patients, we recorded 2887 pDDIs in total (counted with repetitions) based on *MediQ* and *Stockley’s drug interaction checker.* MS—multiple sclerosis; pDDI—potential drug–drug interaction.

The 2887 pDDIs that were identified consisted of mainly mild pDDIs (65.4%) (Figure 2). Mildly moderate pDDIs accounted for 17.7% and moderate pDDIs for 12.9% of all pDDIs. Moderately severe pDDIs made up a rather small percentage of 3.7%. Only 7 of all 2887 pDDIs (0.2%) were rated as severe by both softwares.

**Figure 3:**
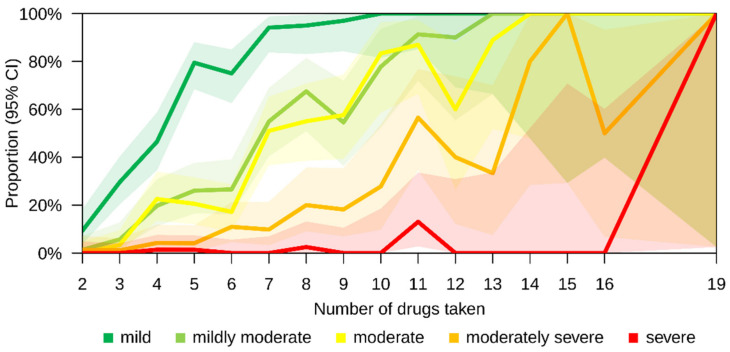
Proportion of patients with MS with at least one pDDI for each severity level and depending on the number of drugs used. The composite rating of pDDI severities was based on *MediQ* and *Stockley’s drug interaction checker*. Both prescription and over-the-counter drugs were considered for this plot. CI—confidence interval; MS—multiple sclerosis; pDDI—potential drug–drug interaction.

Of all 627 patients, 57.9% had at least one mild pDDI, 27.8% had at least one moderate pDDI and 11.0% had at least one moderately severe pDDI (Table 4). For 36.2% of all patients, we found no pDDI at all. Evidently, pDDIs were much more prevalent in PwP than in Pw/oP, independently of the degree of pDDI severity. For total PP, we found that 88.9% of all PwP had at least one mild pDDI, while this applied to only 22.5% of all Pw/oP (Fisher’s exact test: *p* < 0.001). Similar numbers were found for Rx PP. Moreover, more than half of the PwP (52.4% for total PP and 64.9% for Rx PP) had at least one mildly moderate pDDI, while less than 10% of the Pw/oP had at least one mildly moderate pDDI (total PP and Rx PP, Fisher’s exact test: *p* < 0.001 for both) (Table 4). The risk of pDDIs increased with the number of drugs used simultaneously. Patients taking five medications (Rx and OTC) already had a 79.5% risk of having a mild pDDI. While an intake of five or fewer drugs resulted in a risk of having a moderately severe pDDI lower than 10%, the likelihood of having a moderately severe pDDI increased above 20% when 10 or more medications were used (Figure 3).

By examining the prescription status of each drug in a pDDI, we found that 77.3% of all pDDIs consisted of two Rx drugs; 19.0% of one Rx drug and one OTC drug; and 3.7% of two OTC drugs (Table 5). The most frequent pDDI was the interaction between cholecalciferol and magnesium, which was rated as a mild interaction (Table 6). It applied to 5.7% of all patients. Another common pDDI was between acetylsalicylic acid and methylprednisolone, which affected 3.3% of all patients and was rated as a moderate interaction. The seven severe pDDIs detected concerned seven different patients (1.1% of all patients). Remarkably, all of the seven severe pDDIs contained citalopram, which was used in combination with one of the following: ciprofloxacin, doxepin, flecainide, levofloxacin, ondansetron and quetiapine. The pDDI of citalopram and ondansetron was found for two patients. A complete list of all pDDIs is provided in Supplementary Table S3.

Patients with at least one comorbidity from the cardiovascular, neurological, orthopedic or psychiatric group were significantly more likely to have at least one pDDI of any severity than those without a comorbidity from these specific groups (Supplementary Table S2). Of those with a cardiovascular comorbidity (e.g., coronary heart disease), 78.2% had at least one pDDI, compared with 58.4% of those without a cardiovascular comorbidity (Fisher’s exact test: *p* < 0.001). Similar differences in the odds of having at least one pDDI were noted when comparing patients with and without psychiatric comorbidity (85.1% vs. 58.7%), neurological comorbidity (76.9% vs. 61.9%) and orthopedic comorbidity (80.8% vs. 61.4%).

## 4. Discussion

The aim of this study was to determine the extent of PP as well as the prevalence and severity of pDDIs among MS patients. As pDDIs are generally underestimated as a problem by both physicians and patients, it is important to give them more attention, especially in patients with chronic diseases such as MS. For this purpose, we conducted a comprehensive evaluation of the pharmaceutical data of patients with MS with regard to the type and number of drugs taken. PP is a common consequence of the extensive drug therapy often required in chronic diseases, and the higher likelihood of pDDIs resulting from this can lead to detrimental effects. This study is one of the first to evaluate the prevalence and risk of pDDIs in patients with MS and the association between PP and pDDI occurrence.

To our knowledge, there were no previous large-scale studies that addressed the issue of pDDIs in patients with MS until today. We previously evaluated pDDIs in MS patients, but this study was focused on a rather small group of women at childbearing age [31]. Comparisons to other chronic neurological or autoimmune diseases are difficult because the majority of studies dealing with pDDIs and PP were conducted in cohorts of elderly patients or nursing home residents [32,33]. These cohorts therefore have a much higher average age, more comorbidities and thus a higher average number of drugs used, resulting in higher prevalence rates of PP and pDDIs, which makes them hardly comparable to our patient cohort. This demonstrates the necessity of our study, as the issue of pDDIs is rarely discussed in younger and middle-aged patients. There is, however, a study from 2001 on the prevalence of pDDIs in the general population in Sweden, based on records on all prescriptions handed out in Swedish pharmacies [34]. The authors found that 13.6% of all prescriptions included at least one pDDI. If this cohort is considered representative of the general population then we can say, by comparing our results (63.8%) to theirs (13.6%), that patients with MS have an approximately 4.5 times higher chance of having at least one pDDI than the general population.

Of all 2887 pDDIs in our study, only seven were severe (0.2%). All of them contained citalopram as one of the interacting drugs. This suggests that the prescription of citalopram is associated with an increased risk of severe pDDIs as compared to other antidepressants. The frequent occurrence of citalopram in pDDIs—and especially those that are rated as severe—was also documented in a study on pDDIs among patients with dementia [35].

A unique feature of our study are the detailed drug data that were obtained and analyzed. Not only Rx and OTC drugs but also herbal and nutritional supplements were recorded and entered into the interaction software. OTC drugs were included in 22.7% of all pDDIs of our patients. In other studies, non-Rx medications were usually not counted [36] or there was no information on their use because they are often not considered relevant when evaluating pDDIs. However, our data showed that neglecting OTC drugs as part of some patients’ medication plan means that a large number of pDDIs are overlooked. The same applies to medications that were only temporarily taken by the patients, such as antibiotics, which were left out in a Danish study addressing the topic of PP [33]. We included all medications that the patients were taking at the time of the interview, regardless of type and duration of their use. We suggest that OTC drugs, supplements and self-medication should be generally included in the assessment of pDDIs.

The negative effects of PP have been closely studied in the past. PP poses a threat to patients by increasing the risk of avoidable negative outcomes [37,38]. Noncompliance and nonadherence to medication, as well as the risk of adverse drug reactions [39], are issues related to the number of drugs that need to be taken regularly [40,41,42]. PP is positively correlated to the risk of pDDIs [43], thereby provoking a higher number of total pDDIs as well as severe pDDIs. Overall, the quality of life of PwP is negatively influenced by PP [44]. Another consequence of PP and pDDIs are higher costs of healthcare [32,36]. It is estimated that between one and two percent of hospitalizations are caused by pDDIs [24], which is an avoidable financial burden to the healthcare system. In our cohort, we found that about 95% of all pDDIs occurred in patients with total PP, even though those patients made up only around half of all patients. Patients with Rx PP (38.6%) accounted for 88.3% of all pDDIs. This is highly relatable to a Swedish register-based study [45], which found that individuals taking five or more prescription drugs (that is, Rx PP) made up almost 80% of the total acquisition cost of Sweden’s drugs while representing only around 25% of the population. Note that these are only acquisition costs for dispensed prescription drugs, thus hospitalization and costs for any other consequences were not included. Similar circumstances may be assumed in Germany. However, the financial factor is not only a social problem, but also one concerning the individual. Some recommended drugs are not covered by the healthcare system but need to be covered by the patients themselves. These are expenses for OTC medications and copayments for prescribed medications. Hence, with the rising number of drugs, costs for patients are rising as well.

A feared consequence of pDDIs that can make them so dangerous is the altered efficacy of one or both drugs interacting, thereby provoking treatment failure [23]. This could be life-threatening to the patient [46]. A change in the drug’s effect and effectiveness can be triggered by another drug by influencing the patient’s metabolism pharmacokinetically and/or pharmacodynamically. Moreover, the drug’s toxicity could be altered and dangerously increased, or new side effects resulting from the combination of two or more drugs might appear. As an example, one of the most frequently recorded pDDIs in our cohort with a frequency of 3.3% was the interaction between acetylsalicylic acid and enoxaparin. The severity of this pDDI was rated as moderate because of an insignificantly increased bleeding risk due to this combination [47]. Such combination side effects that can cause new complaints may even result in the prescription of additional drugs, leading to a prescribing cascade [48].

By reducing the number of drugs used, PP rates as well as the number of pDDIs will also decrease. To achieve this, there are several approaches for doctors and healthcare providers, including pharmacists. The simplest one is to deprescribe any unnecessary medication and to stop prescribing new medication that may not be necessary. Unnecessary medications can be those that do not contribute significantly to the patients’ health, that have the same active agents as one of the other drugs the patient is taking or even OTC drugs that are more likely to do harm than good. Another way is to regularly check each patient’s medication, for example, in the form of a “brown bag review” [49], which has been found to be well-working [50]. Following this approach, the patient is asked to bring all drugs currently being taken, including self-bought OTC drugs and nutritional or herbal supplements. This might give the doctor a better overview of what the patient is actually taking—especially supplements and OTC drugs, which are otherwise often not considered [51,52]. Another similar approach is called medication therapy management (MTM), a service provided by participating pharmacies that was implemented by the American Pharmacists Association and several national pharmacy groups from the US. MTM includes a wide range of services such as medication therapy review, a personal medication record and a medication-related action plan, aiming at improving therapeutic outcomes and reducing drug-related problems [53]. Studies have found MTM to improve clinical outcomes, medication adherence and appropriateness [54,55]. It also entails economic advantages by reducing healthcare costs [56]. These results show that a national implementation of this kind of service in local pharmacies could reduce the number of drugs taken, and therefore the number of pDDIs and the number of inpatient admissions. Approaches such as this one should be emphasized. In general, the role of pharmacists as the experts for medication should not be forgotten, as they are an important link between doctors and patients. It has been shown that pharmacists’ interventions can be an effective factor in identifying and managing pDDIs, as they can intervene on different levels [57], for example, by contacting the prescriber and discussing critical drug combinations or new prescriptions, by changing the dosage or formulation of a drug after consulting the doctor or by advising the patient and giving instructions on how to prevent and detect pDDIs (i.e., more frequent blood glucose monitoring at home). Of course, this does not only apply to Rx medications but also to OTCs and dietary supplements, for which counseling might be even more important, as doctors mostly inform their patients less about these than compared with Rx drugs. Another outstanding advantage pharmacists usually have is the overview of all prescriptions even from different specialist doctors, whereas doctors mostly only see the drugs they are prescribing. For all of this, a trustful patient–pharmacist relationship is important [58], which can therefore be a limiting factor: if a patient does not have one pharmacy of trust, but uses several pharmacies at the same time, it might be more difficult to build a relationship to one pharmacist and the advantage of a medication schedule overview becomes lost, resulting in a less adequate consultation.

Furthermore, when prescribing a new drug, doctors should always check for pDDIs with the current medication. In addition, the connection between doctors and pharmacists should be strengthened, so that doctors can consult pharmacists more regularly to discuss patients’ medication schedule and pharmacists can advise doctors about, for example, newly approved drugs. Further, regular checkups with CDSS should be carried out as they are updated occasionally. Hence, doctors should make it a routine to check each patient’s medication plan (for example, once a year). Pharmacists should always give advice about possible complications and ask for existing medication to check for possible interactions when handing out new substances (Rx as well as OTC drugs). Another important factor is treatment adherence, which also ought to be checked regularly. If patients do not take their medication properly, doctors may be tempted to prescribe new medications because therapeutic effects are not apparent. Another way to interrupt possible prescribing cascades is to provide a medication plan for each patient, where all their drugs are listed with indication and dosage. This gives a better overview for the patient as well as for other doctors the patient is seeing. This is especially true since, in a disease with a “thousand different faces”, doctors from different disciplines may be involved in the treatment of the patient with MS. By knowing what other drugs the patient is taking, doctors can pursue possible causes of side effects that they might otherwise mistakenly consider as separate illnesses. For example, if a patient is taking opioid analgesics, their doctor would know that possible constipation is not an independent symptom but a side effect of the established medication [44]. There are many more approaches to reduce the risk of PP and of possibly dangerous DDIs [59,60].

With an average age of 48.6 years, our cohort was similar to two large national MS cohorts with a mean age of 49 years [61] and 46 years [62], respectively. The same holds for the mean age at disease onset. According to the atlas of MS, mean age at MS onset in Germany is 33 years [4], while in our cohort, it was at 35 years (Table 1). The sex ratio in our study cohort of around 2.4 to 1 (female vs. male) was also highly similar to that reported in other studies [61,63,64]. Our data on PP and pDDIs are thus based on a representative study population of 627 patients with CIS/MS in total.

Nevertheless, there are limitations of our study. As it is a cross-sectional study, no causal relationships can be derived. However, several associations could be clearly shown. These should be studied in more detail in longitudinal studies. Moreover, as the data acquisition was performed by conducting patient interviews, an underestimation of the number of drugs taken by the patients is possible, especially with regard to the use of OTC drugs and nutritional supplements. Studies have found discrepancies regarding patient-reported medication plans and medical records: up to 60% of patients had at least one omission error in what they reported to take [65], which means that they did not report at least one drug that they used before hospital admission, where the interview took place. The rate of falsely reported drugs is particularly high for the medication group of nonsteroid anti-inflammatory drugs (NSAIDs) [65,66]. Especially for OTC NSAIDs, such as ibuprofen or diclofenac, omission errors are frequent [66]. Therefore, we presume unreported drugs and therefore undetected pDDIs in our data. The same applies to compliance and adherence, as we do not know if the patients really took their medication properly as prescribed. Further, metabolism is different in each patient, and many of the interactions that were detected in the CDSS and DDID are dependent on, e.g., dosage, metabolic factors, application form and timing of the concurrently taken drugs. As many pDDIs are mediated through induction or inhibition of cytochrome P450 (CYP) enzymes, interindividual differences in the expression of these enzymes make it difficult to predict a patient’s reaction to drugs [67,68]. For this reason, we are referring to *potential* DDIs. It cannot be concluded that any of these are guaranteed to apply, but there is always the possibility that the pDDI will actually occur. What should be further investigated is the question whether patients actually know about the risk of pDDIs and whether they are informed by their doctors about warning signs and symptoms of pDDIs.

It should be noted that every CDSS or DDID has a different definition of, for example, a “mild” interaction and the classification of pDDI severities is difficult in general. Moreover, each CDSS/DDID also has some limitations. In *MediQ*, some substances, especially nutritional supplements and homeopathics, were not available, such as vitamin K2 or canephron. Conversely, in *Stockley’s,* the information was not as precise as in *MediQ* at some points, as for example, different hormonal substances were summarized under the term “oral contraceptives”, no matter which estrogens or gestagens were entered.

## 5. Conclusions

To conclude, we found that 53.3% of the studied patients with MS had total PP. For 63.8% of our patients, we detected at least one pDDI. The majority (65.4%) of all recorded pDDIs was rated as mild. pDDIs occurred significantly more often in patients with total PP (93.4%) and patients with Rx PP (97.1%) than in those without PP. This underlines that PP is a significant predictor of pDDIs [69]. Comorbidities were related to the occurrence of at least one pDDI: significantly higher pDDI prevalence rates were found for patients with cardiovascular, neurological, psychiatric and orthopedic comorbidities. Although only a small percentage (1.1%) of patients had a severe pDDI, representing a direct threat to the patients, the overall number of pDDIs was surprisingly high. Our study shows that there is a need for more awareness of PP and pDDIs. Doctors in general, but especially those treating chronically ill patients such as those with MS, should pay special attention to pDDIs. The use of CDSS/DDID to detect pDDIs may help avoiding them and should be integrated in physicians’ daily routines. Doctors should be informed about their patients’ OTC drug use and include these OTC drugs in their evaluation of pDDIs. Future studies should focus on PP and pDDIs in middle-aged patients and further uncover possible dangers in patient healthcare that can be avoided.

## Figures and Tables

**Table 1:** Sociodemographic, clinical and pharmaceutical data of patients with MS, stratified by polypharmacy status.

			Total Polypharmacy					Rx Polypharmacy				
	All Patients		PwP		Pw/oP		*p*	PwP		Pw/oP		*p*
**N**	627		334 (53.3%)		293 (46.7%)			242 (38.6%)		385 (61.4%)		
**Sociodemographic data**												
**Sex**							0.793 ^Fi^					0.720 ^Fi^
Male	186 (29.7%)		101 (30.2%)		85 (29.0%)			74 (30.6%)		112 (29.1%)		
Female	441 (70.3%)		233 (69.8%)		208 (71.0%)			168 (69.4%)		273 (70.9%)		
**Age (years)**	19–86 ^R^	48.6 (13.3) ^a^	20–86 ^R^	53.0 (12.7) ^a^	19–74 ^R^	43.6 (12.2) ^a^	**<0.001 ^t^**	24–86 ^R^	54.8 (12.1) ^a^	19–75 ^R^	44.7 (12.5) ^a^	**<0.001 ^t^**
**School years**	6–18 ^R^	10.5 (1.3) ^a^	6–14 ^R^	10.3 (1.2) ^a^	8–18 ^R^	10.7 (1.3) ^a^	**<0.001 ^t^**	6–14 ^R^	10.2 (1.2) ^a^	8–18 ^R^	10.7 (1.3) ^a^	**<0.001 ^t^**
**Educational level**							**0.019 ^Chi^**					**0.002 ^Chi^**
No training	19 (3.0%)		8 (2.4%)		11 (3.8%)			7 (2.9%)		12 (3.1%)		
Skilled worker	398 (63.5%)		229 (68.6%)		169 (57.7%)			173 (71.5%)		225 (58.4%)		
Technical college	89 (14.2%)		46 (13.8%)		43 (14.7%)			33 (13.6%)		56 (14.5%)		
University	121 (19.3%)		51 (15.3%)		70 (23.9%)			29 (12.0%)		92 (23.9%)		
**Employment status**							**<0.001 ^Chi^**					**<0.001 ^Chi^**
In training	7 (1.1%)		1 (0.3%)		6 (2.0%)			0 (0.0%)		7 (1.8%)		
In studies	6 (1.0%)		0 (0.0%)		6 (2.0%)			0 (0.0%)		6 (1.6%)		
Employed	269 (42.9%)		92 (27.5%)		177 (60.4%)			53 (21.9%)		216 (56.1%)		
Unemployed	25 (4.0%)		10 (3.0%)		15 (5.1%)			7 (2.9%)		18 (4.7%)		
Disability-pensioned	304 (48.5%)		225 (67.4%)		79 (27.0%)			178 (73.6%)		126 (32.7%)		
Other	16 (2.6%)		6 (1.8%)		10 (3.4%)			4 (1.7%)		12 (3.1%)		
**Partnership**							1.000 ^Fi^					0.305 ^Fi^
No	162 (25.8%)		86 (25.7%)		76 (25.9%)			68 (28.1%)		94 (24.4%)		
Yes	465 (74.2%)		248 (74.3%)		217 (74.1%)			174 (71.9%)		291 (75.6%)		
**Place of Residence**							0.288 ^Chi^					0.962 ^Chi^
Rural community	224 (35.7%)		119 (35.6%)		105 (35.8%)			89 (36.8%)		135 (35.1%)		
Provincial town	108 (17.2%)		63 (18.9%)		45 (15.4%)			42 (17.4%)		66 (17.1%)		
Medium-sized town	112 (17.9%)		64 (19.2%)		48 (16.4%)			43 (17.8%)		69 (17.9%)		
City	183 (29.2%)		88 (26.3%)		95 (32.4%)			68 (28.1%)		115 (29.9%)		
**Number of children**	0–4 ^R^	1 ^b^	0–4 ^R^	1 ^b^	0–4 ^R^	1 ^b^	0.089 ^U^	0–4 ^R^	1 ^b^	0–4 ^R^	1 ^b^	0.056 ^U^
0	169 (27.0%)		77 (23.1%)		92 (31.4%)			54 (22.3%)		115 (29.9%)		
1	170 (27.1%)		98 (29.3%)		72 (24.6%)			68 (28.1%)		102 (26.5%)		
≥2	288 (45.9%)		159 (47.6%)		129 (44.0%)			120 (49.6%)		168 (43.6%)		
**Number of siblings**	0–13 ^R^	1 ^b^	0–13 ^R^	1 ^b^	0–11 ^R^	1 ^b^	0.081 ^U^	0–13 ^R^	1 ^b^	0–11 ^R^	1 ^b^	**0.018 ^U^**
0	71 (11.3%)		33 (9.9%)		38 (13.0%)			26 (10.7%)		45 (11.7%)		
1	305 (48.6%)		160 (47.9%)		145 (49.5%)			103 (42.6%)		202 (52.5%)		
≥2	251 (40.0%)		141 (42.2%)		110 (37.5%)			113 (46.7%)		138 (35.8%)		
**Clinical data**												
**EDSS**	0–9 ^R^	3.5 ^b^	0–9 ^R^	4.5 ^b^	0–7.5 ^R^	2.0 ^b^	**<0.001 ^U^**	0–9 ^R^	5.0 ^b^	0–7.5 ^R^	2.5 ^b^	**<0.001 ^U^**
**Disease duration (years)**	0–52 ^R^	10 ^b^	0–50 ^R^	12.5 ^b^	0–52 ^R^	9 ^b^	**<0.001 ^U^**	0–50 ^R^	14 ^b^	0–52 ^R^	9 ^b^	**<0.001 ^U^**
**Age at MS onset**	9–75 ^R^	35 ^b^	9–75 ^R^	38 ^b^	12–62 ^R^	32 ^b^	**<0.001 ^U^**	9–75 ^R^	39 ^b^	12–69 ^R^	33 ^b^	**<0.001 ^U^**
**Disease course**							**<0.001 ^Chi^**					**<0.001 ^Chi^**
CIS/RRMS	415 (66.2%)		158 (47.3%)		257 (87.7%)			91 (37.6%)		324 (84.2%)		
SPMS	154 (24.6%)		125 (37.4%)		29 (9.9%)			109 (45.0%)		45 (11.7%)		
PPMS	58 (9.3%)		51 (15.3%)		7 (2.4%)			42 (17.4%)		16 (4.2%)		
**Comorbidities**	0–9 ^R^	1 ^b^	0–9 ^R^	2 ^b^	0–5 ^R^	1 ^b^	**<0.001 ^U^**	0–9 ^R^	3 ^b^	0–7 ^R^	1 ^b^	**<0.001 ^U^**
0	184 (29.3%)		46 (13.8%)		138 (47.1%)			24 (9.9%)		160 (41.6%)		
1	150 (23.9%)		60 (18.0%)		90 (30.7%)			39 (16.1%)		111 (28.8%)		
2	122 (19.5%)		76 (22.8%)		46 (15.7%)			50 (20.7%)		72 (18.7%)		
3	82 (13.1%)		71 (21.3%)		11 (3.8%)			58 (24.0%)		24 (6.2%)		
4	50 (8.0%)		44 (13.2%)		6 (2.0%)			35 (14.5%)		15 (3.9%)		
≥5	39 (6.2%)		37 (11.1%)		2 (0.7%)			36 (14.9%)		3 (0.8%)		
**Pharmaceutical data**												
**Number of total drugs taken**	0–19 ^R^	5.3 (3.3) ^c^	5–19 ^R^	7.8 (2.7) ^c^	0–4 ^R^	2.6 (1.1) ^c^	**<0.001 ^t^**	5–19 ^R^	8.5 (2.7) ^c^	0–9 ^R^	3.3 (1.7) ^c^	**<0.001 ^t^**
0–4	293 (46.7%)		0 (0.0%)		293 (100.0%)			0 (0.0%)		293 (76.1%)		
5–9	261 (41.6%)		261 (78.1%)		0 (0.0%)			169 (69.8%)		92 (23.9%)		
≥10	73 (11.6%)		73 (21.9%)		0 (0.0%)			73 (30.2%)		0 (0.0%)		
**Duration of use**												
Long-term drugs	0–16 ^R^	4.6 (3.1) ^c^	1–16 ^R^	6.7 (2.7) ^c^	0–4 ^R^	2.2 (1.1) ^c^	**<0.001 ^t^**	1–16 ^R^	7.4 (2.7) ^c^	0–9 ^R^	2.8 (1.7) ^c^	**<0.001 ^t^**
PRN drugs	0–7 ^R^	0.8 (1.2) ^c^	0–7 ^R^	1.1 (1.4) ^c^	0–4 ^R^	0.4 (0.7) ^c^	**<0.001 ^t^**	0–7 ^R^	1.2 (1.4) ^c^	0–6 ^R^	0.6 (0.9) ^c^	**<0.001 ^t^**
**Rx vs. OTC**												
Rx drugs	0–18 ^R^	4.2 (3.0) ^c^	1–18 ^R^	6.2 (2.8) ^c^	0–4 ^R^	1.9 (1.0) ^c^	**<0.001 ^t^**	5–18 ^R^	7.3 (2.4) ^c^	0–4 ^R^	2.2 (1.2) ^c^	**<0.001 ^t^**
OTC drugs	0–8 ^R^	1.1 (1.3) ^c^	0–8 ^R^	1.6 (1.4) ^c^	0–3 ^R^	0.6 (0.8) ^c^	**<0.001 ^t^**	0–6 ^R^	1.2 (1.3) ^c^	0–8 ^R^	1.1 (1.3) ^c^	0.206 ^t^
**Drug purpose**												
DMD	0–2 ^R^	0.9 (0.4) ^c^	0–2 ^R^	0.9 (0.4) ^c^	0–2 ^R^	0.8 (0.4) ^c^	**0.004 ^t^**	0–2 ^R^	0.9 (0.4) ^c^	0–2 ^R^	0.8 (0.4) ^c^	**<0.001 ^t^**
Symptomatic drugs	0–9 ^R^	2.0 (2.0) ^c^	0–9 ^R^	3.1 (2.0) ^c^	0–3 ^R^	0.7 (0.9) ^c^	**<0.001 ^t^**	0–9 ^R^	3.3 (2.0) ^c^	0–9 ^R^	1.2 (1.4) ^c^	**<0.001 ^t^**
Comorbidity drugs	0–14 ^R^	2.5 (2.4) ^c^	0–14 ^R^	3.8 (2.6) ^c^	0–4 ^R^	1.0 (0.9) ^c^	**<0.001 ^t^**	0–14 ^R^	4.3 (2.7) ^c^	0–7 ^R^	1.3 (1.3) ^c^	**<0.001 ^t^**

Total polypharmacy = intake of at least five drugs (of any kind). Rx polypharmacy = intake of at least five drugs that were prescribed (neglecting OTC drugs). ^a^—mean value (standard deviation); ^b^—median; ^c^—average number of drugs taken per patient (standard deviation); ^Chi^—chi-squared test; CIS—clinically isolated syndrome; DMD—disease-modifying drug; EDSS—Expanded Disability Status Scale; ^Fi^—Fisher’s exact test; MS—multiple sclerosis; N—number of patients; OTC—over the counter; *p—p*-value for comparing patients with and without polypharmacy; PwP—patients with polypharmacy; Pw/oP—patients without polypharmacy; PPMS—primary progressive multiple sclerosis; PRN—*pro re nata* (on demand); R—range; RRMS—relapsing–remitting multiple sclerosis; Rx—prescription; ^t^—two-sample two-tailed Welch *t*-test; ^U^—Mann–Whitney U test.

**Table 2:** Total number of all recorded medications (counted with repetitions), subdivided by drug category and polypharmacy status of the patients with MS (N = 627).

		Total Polypharmacy			Rx Polypharmacy		
Drug Category	Total Number of Drugs	PwP	Pw/oP	*p*	PwP	Pw/oP	*p*
**All**	3341 (100%)	2591 (77.6%)	750 (22.4%)		2060 (61.7%)	1281 (38.3%)	
**Duration of use**				0.176 ^Fi^			**0.013 ^Fi^**
Long-term drugs	2855 (85.5%)	2226 (85.9%)	629 (83.9%)		1785 (86.7%)	1070 (83.5%)	
PRN drugs	486 (14.5%)	365 (14.1%)	121 (16.1%)		275 (13.3%)	211 (16.5%)	
**Rx vs. OTC**				**0.011 ^Fi^**			<**0.001 ^Fi^**
Rx drugs	2630 (78.7%)	2065 (79.7%)	565 (75.3%)		1766 (85.7%)	864 (67.4%)	
OTC drugs	711 (21.3%)	526 (20.3%)	185 (24.7%)		294 (14.3%)	417 (32.6%)	
**Drug purpose**				<**0.001 ^Chi^**			<**0.001 ^Chi^**
DMD	530 (15.9%)	297 (11.5%)	233 (31.1%)		223 (10.8%)	307 (24.0%)	
Symptomatic drugs	1253 (37.5%)	1035 (39.9%)	218 (29.0%)		796 (38.6%)	457 (35.7%)	
Comorbidity drugs	1558 (46.6%)	1259 (48.6%)	299 (39.9%)		1041 (50.6%)	517 (40.3%)	

Total polypharmacy = intake of at least five drugs (of any kind). Rx polypharmacy = intake of at least five drugs that were prescribed (neglecting OTC drugs). ^Chi^—chi-squared test; DMD—disease-modifying drug; MS—multiple sclerosis; OTC—over the counter; *p*—*p*-value for comparing patients with and without polypharmacy; PwP—patients with polypharmacy; Pw/oP—patients without polypharmacy; PRN—*pro re nata* (on demand); Rx—prescription.

**Table 3:** Most frequently used non-DMDs and DMDs among MS patients with and without polypharmacy.

		Total Polypharmacy			Rx Polypharmacy		
	All Patients	PwP	Pw/oP	*p* ^Fi^	PwP	Pw/oP	*p* ^Fi^
**N**	627	334 (53.3%)	293 (46.7%)		242 (38.6%)	385 (61.4%)	
**Most used non-DMDs**							
Cholecalciferol	261 (41.6%)	178 (53.3%)	83 (28.3%)	**<0.001**	125 (51.7%)	136 (35.3%)	**<0.001**
Pantoprazole	178 (28.4%)	155 (46.4%)	23 (7.8%)	**<0.001**	144 (59.5%)	34 (8.8%)	**<0.001**
Enoxaparin	127 (20.3%)	114 (34.1%)	13 (4.4%)	**<0.001**	105 (43.3%)	22 (5.7%)	**<0.001**
Ibuprofen	105 (16.7%)	61 (18.3%)	44 (15.0%)	0.286	41 (16.9%)	64 (16.6%)	0.913
Baclofen	78 (12.4%)	72 (21.6%)	6 (2.0%)	**<0.001**	68 (28.1%)	10 (2.6%)	**<0.001**
Levothyroxine	75 (12.0%)	51 (15.3%)	24 (8.2%)	**0.007**	41 (16.9%)	34 (8.8%)	**0.003**
Cyanocobalamin	66 (10.5%)	46 (13.8%)	20 (6.8%)	**0.006**	27 (11.2%)	39 (10.1%)	0.690
Zopiclone	65 (10.4%)	58 (17.4%)	7 (2.4%)	**<0.001**	53 (21.9%)	12 (3.1%)	**<0.001**
Magnesium	60 (9.6%)	45 (13.5%)	15 (5.1%)	**<0.001**	21 (8.7%)	39 (10.1%)	0.580
Acetylsalicylic acid	55 (8.8%)	48 (14.4%)	7 (2.4%)	**<0.001**	41 (16.9%)	14 (3.6%)	**<0.001**
**DMDs (all, incl. methylprednisolone)**							
Methylprednisolone	123 (19.6%)	110 (32.9%)	13 (4.4%)	**<0.001**	101 (41.7%)	22 (5.7%)	**<0.001**
Interferon beta-1a	64 (10.2%)	25 (7.5%)	39 (13.3%)	**0.018**	14 (5.8%)	50 (13.0%)	**0.004**
Glatiramer acetate	57 (9.1%)	21 (6.3%)	36 (12.3%)	**0.012**	14 (5.8%)	43 (11.2%)	**0.023**
Natalizumab	47 (7.5%)	18 (5.4%)	29 (9.9%)	**0.034**	9 (3.7%)	38 (9.9%)	**0.005**
Fingolimod	41 (6.5%)	21 (6.3%)	20 (6.8%)	0.872	15 (6.2%)	26 (6.8%)	0.869
Teriflunomide	36 (5.7%)	19 (5.7%)	17 (5.8%)	1.000	11 (4.5%)	25 (6.5%)	0.379
Dimethyl fumarate	32 (5.1%)	10 (3.0%)	22 (7.5%)	**0.011**	8 (3.3%)	24 (6.2%)	0.135
Mitoxantrone	28 (4.5%)	15 (4.5%)	13 (4.4%)	1.000	11 (4.5%)	17 (4.4%)	1.000
Ocrelizumab	27 (4.3%)	25 (7.5%)	2 (0.7%)	**<0.001**	19 (7.9%)	8 (2.1%)	**0.001**
Interferon beta-1b	23 (3.7%)	9 (2.7%)	14 (4.8%)	0.203	7 (2.9%)	16 (4.2%)	0.515
Alemtuzumab	20 (3.4%)	5 (1.5%)	15 (5.1%)	**0.012**	2 (0.8%)	18 (4.7%)	**0.009**
Immunoglobulin G	7 (1.1%)	3 (0.3%)	4 (1.4%)	0.711	0 (0.0%)	7 (1.8%)	**0.047**
Cladribine	6 (1.0%)	2 (0.6%)	4 (1.4%)	0.426	2 (0.8%)	4 (1.0%)	1.000
Azathioprine	4 (0.6%)	2 (0.6%)	2 (0.7%)	1.000	1 (0.4%)	3 (0.8%)	1.000
Rituximab	2 (0.3%)	1 (0.3%)	1 (0.3%)	1.000	0 (0.0%)	2 (0.5%)	0.525

Total polypharmacy = intake of at least five drugs (of any kind). Rx polypharmacy = intake of at least five drugs that were prescribed (neglecting OTC drugs). DMD—disease-modifying drug for the treatment of MS; Fi—Fisher’s exact test; MS—multiple sclerosis; N—number of patients; *p*—*p*-value for comparing patients with and without polypharmacy; PwP—patients with polypharmacy; Pw/oP—patients without polypharmacy; Rx—prescription.

**Table 4:** Prevalence of pDDIs in the patients with MS.

		Total Polypharmacy			Rx Polypharmacy		
	All Patients	PwP	Pw/oP	*p* ^Fi^	PwP	Pw/oP	*p* ^Fi^
**N**	627	334 (53.3%)	293 (46.7%)		242 (38.6%)	385 (61.4%)	
**Severity level**							
**Mild**	363 (57.9%)	297 (88.9%)	66 (22.5%)	**<0.001**	225 (93.0%)	138 (35.8%)	**<0.001**
**Mildly moderate**	195 (31.1%)	175 (52.4%)	20 (6.8%)	**<0.001**	157 (64.9%)	38 (9.9%)	**<0.001**
**Moderate**	174 (27.8%)	155 (46.4%)	19 (6.5%)	**<0.001**	140 (57.9%)	34 (8.8%)	**<0.001**
**Moderately severe**	69 (11.0%)	64 (19.2%)	5 (1.7%)	**<0.001**	61 (25.2%)	8 (2.1%)	**<0.001**
**Severe**	7 (1.1%)	6 (1.8%)	1 (0.3%)	0.129	5 (2.1%)	2 (0.5%)	0.114
**No pDDI at all**	227 (36.2%)	22 (6.6%)	205 (70.0%)	**<0.001**	7 (2.9%)	220 (57.1%)	**<0.001**

Each patient who had at least one pDDI of a given category was counted. Note that the patients could have several pDDIs of different severities at the same time. The level of severity of pDDIs was assessed based on *MediQ* and *Stockley’s drug interaction checker*. Total polypharmacy = intake of at least five drugs (of any kind). Rx polypharmacy = intake of at least five drugs that were prescribed (neglecting OTC drugs). ^Chi^—chi-squared test; ^Fi^—Fisher’s exact test; MS—multiple sclerosis; N— number of patients; *p*—*p*-value for comparing patients with and without polypharmacy; pDDI—potential drug–drug interaction; PwP—patients with polypharmacy; Pw/oP—patients without polypharmacy; Rx—prescription.

**Table 5:** Distribution of pDDIs depending on severity level and prescription.

	Total Number of pDDIs Recorded	Rx-Rx	Rx-OTC	OTC-OTC	*p* ^Chi^
**N**	2887	2231 (77.3%)	549 (19.0%)	107 (3.7%)	
**Severity level**					**<0.001**
**Mild**	1889 (65.4%)	1469 (65.8%)	327 (59.6%)	93 (86.9%)	
**Mildly moderate**	511 (17.7%)	417 (18.7%)	85 (15.5%)	9 (8.4%)	
**Moderate**	373 (12.9%)	249 (11.2%)	120 (21.9%)	4 (3.7%)	
**Moderately severe**	107 (3.7%)	89 (4.0%)	17 (3.1%)	1 (0.9%)	
**Severe**	7 (0.2%)	7 (0.3%)	0 (0.0%)	0 (0.0%)	

The pDDIs were categorized by severity and whether they contained Rx and/or OTC drugs. The level of severity of pDDIs was assessed based on *MediQ* and *Stockley’s* drug interaction checker. Rx-Rx, Rx-OTC and OTC-OTC relate to the kind of drugs the pDDI was made up of. ^Chi^—chi-squared test; N—number of pDDIs; OTC—over the counter; *p—p*-value for comparing the proportions of the different pDDI categories; pDDI—potential drug–drug interaction; Rx—prescription.

**Table 6:** The most common pDDIs in patients with MS.

			Total Amount (N = 627)	Total Polypharmacy		Rx Polypharmacy	
Drug 1	Drug 2	pDDI Severity		Amount in PwP (N = 334)	Amount in Pw/oP (N = 293)	Amount in PwP (N = 242)	Amount in Pw/oP (N = 385)
**pDDIs of non-DMDs**							
Cholecalciferol	Magnesium	mild	36 (5.7%)	30 (9.0%)	6 (2.0%)	15 (6.2%)	21 (5.5%)
Cyanocobalamin	Pantoprazole	mild	27 (4.3%)	25 (7.5%)	2 (0.7%)	23 (9.5%)	4 (1.0%)
Calcium	Cholecalciferol	mild	26 (4.1%)	25 (7.5%)	1 (0.3%)	22 (9.1%)	4 (1.0%)
Levothyroxine	Pantoprazole	mildly moderate	23 (3.7%)	22 (6.6%)	1 (0.3%)	22 (9.1%)	1 (0.3%)
Acetylsalicylic acid	Enoxaparin	moderate	21 (3.3%)	20 (6.0%)	1 (0.3%)	19 (7.9%)	2 (0.5%)
Cholecalciferol	Simvastatin	mild	20 (3.2%)	19 (5.7%)	1 (0.3%)	19 (7.9%)	1 (0.3%)
Baclofen	Fampridine	mild	20 (3.2%)	19 (5.7%)	1 (0.3%)	17 (7.0%)	3 (0.8%)
Cholecalciferol	Prednisolone	mild	18 (2.9%)	18 (5.4%)	0 (0.0%)	15 (6.2%)	3 (0.8%)
Pantoprazole	Torasemide	mild	18 (2.9%)	18 (5.4%)	0 (0.0%)	18 (7.4%)	0 (0.0%)
Cyanocobalamin	Folic acid	mild	17 (2.7%)	12 (3.6%)	5 (1.7%)	8 (3.3%)	9 (2.3%)
**pDDIs of DMDs incl. methylprednisolone**							
Acetylsalicylic acid	Methylprednisolone	moderate	21 (3.3%)	20 (6.0%)	1 (0.3%)	19 (7.9%)	2 (0.5%)
Ibuprofen	Methylprednisolone	mildly moderate	14 (2.2%)	13 (3.9%)	1 (0.3%)	13 (5.4%)	1 (0.3%)
Methylprednisolone	Ramipril	mild	12 (1.9%)	12 (3.6%)	0 (0.0%)	12 (5.0%)	0 (0.0%)
Citalopram	Methylprednisolone	moderately severe	10 (1.6%)	10 (3.0%)	0 (0.0%)	10 (4.1%)	0 (0.0%)
Methylprednisolone	Torasemide	mild	10 (1.6%)	10 (3.0%)	0 (0.0%)	10 (4.1%)	0 (0.0%)
Dipyrone	Methylprednisolone	moderate	9 (1.4%)	9 (2.7%)	0 (0.0%)	9 (3.7%)	0 (0.0%)
Methylprednisolone	Solifenacin	mildly moderate	9 (1.4%)	9 (2.7%)	0 (0.0%)	8 (3.3%)	1 (0.3%)
Citalopram	Fingolimod	moderately severe	7 (1.1%)	5 (1.5%)	2 (0.7%)	3 (1.2%)	4 (1.0%)
Mitoxantrone	Ondansetron	mildly moderate	7 (1.1%)	4 (1.2%)	3 (1.0%)	3 (1.2%)	4 (1.0%)
Interferon beta-1a	Ramipril	mildly moderate	7 (1.1%)	5 (1.5%)	2 (0.7%)	4 (1.7%)	3 (0.8%)

Shown are the 10 most frequently detected interactions with and without the involvement of DMDs. The level of severity of pDDIs was assessed based on *MediQ* and *Stockley’s* drug interaction checker. Total polypharmacy = intake of at least five drugs (of any kind). Rx polypharmacy = intake of at least five drugs that were prescribed (neglecting OTC drugs). DMD—disease-modifying drug for the treatment of MS; MS—multiple sclerosis; N—number of patients; pDDI—potential drug–drug interaction; PwP—patients with polypharmacy; Pw/oP—patients without polypharmacy; Rx—prescription.

## Data Availability

The data supporting the findings of this study are available in the article and/or Supplementary Materials. Readers are welcome to contact the corresponding author by e-mail to request access to the raw de-identified data used in this work.

## Supplementary Materials

The following supporting information can be downloaded at: https://www.mdpi.com/article/10.3390/pharmaceutics14030592/s1, Table S1: Groups of comorbidities present in MS patients with and without polypharmacy, Table S2: Prevalence of pDDIs in MS patients stratified by different comorbidity groups, Table S3: All pDDIs of all patients with MS.
