# Supplementary material for: Prevalence and Severity of Potential Drug–Drug Interactions in Patients with Multiple Sclerosis with and without Polypharmacy
Source: Pharmaceutics. 2022 Mar 8;14(3):592. doi: 10.3390/pharmaceutics14030592 (PMC8949310; doi:10.3390/pharmaceutics14030592)
Supplement: Supplementary file 1 [file pharmaceutics-14-00592-s001.zip › Supplement Table S1.pdf]

Supplement Table S1. Groups of comorbidities present in MS patients with and without polypharmacy.

		Total polypharmacy			Rx polypharmacy		
	All patients	PwP	Pw/oP	p^{Fi}	PwP	Pw/oP	p^{Fi}
N	627	334 (53.3%)	293 (46.7%)		242 (38.6%)	385 (61.4%)	
**Comorbidity groups**							
**Cardiovascular**	170 (27.1%)	108 (32.3%)	62 (21.2%)	**0.002**	89 (36.8%)	81 (21.0%)	**<0.001**
**Psychiatric**	121 (19.3%)	86 (25.7%)	35 (11.9%)	**<0.001**	71 (29.3%)	50 (13.0%)	**<0.001**
**Metabolic**	111 (17.7%)	70 (21.0%)	41 (14.0%)	**0.027**	59 (24.4%)	52 (13.5%)	**0.001**
**Neurological**	78 (12.4%)	55 (16.5%)	23 (7.8%)	**0.001**	46 (19.0%)	32 (8.3%)	**<0.001**
**Orthopedic**	78 (12.4%)	58 (17.4%)	20 (6.8%)	**<0.001**	47 (19.4%)	31 (8.1%)	**<0.001**
**Gastrointestinal**	66 (10.5%)	45 (13.5%)	21 (7.2%)	**0.013**	36 (14.9%)	30 (7.8%)	**0.007**
**Urological/ gynecological**	51 (8.1%)	33 (9.9%)	18 (6.1%)	0.107	26 (10.7%)	25 (6.5%)	0.071
**Endocrinological**	49 (7.8%)	26 (7.8%)	23 (7.8%)	1.000	19 (7.9%)	30 (7.8%)	1.000
**Pulmonary**	27 (4.3%)	14 (4.2%)	13 (4.4%)	1.000	14 (5.8%)	13 (3.4%)	0.161
**Dermatological**	23 (3.7%)	11 (3.3%)	12 (4.1%)	0.672	10 (4.1%)	13 (3.4%)	0.665
**Ophtalmological**	23 (3.7%)	19 (5.7%)	4 (1.4%)	**0.005**	17 (7.0%)	6 (1.6%)	**0.001**
**Chronic inflammatory**	11 (1.8%)	6 (1.8%)	5 (1.7%)	1.000	5 (2.1%)	6 (1.6%)	0.757
**Haematological**	7 (1.1%)	4 (1.2%)	3 (1.0%)	1.000	2 (0.8%)	5 (1.3%)	0.712
**Ear-nose-throat**	6 (1.0%)	4 (1.2%)	2 (0.7%)	0.690	3 (1.2%)	3 (0.8%)	0.681
**Pain**	5 (0.8%)	2 (0.6%)	3 (1.0%)	0.669	2 (0.8%)	3 (0.8%)	1.000
**Others**	68 (10.8%)	43 (12.9%)	25 (8.5%)	0.094	32 (13.2%)	36 (9.4%)	0.147

Any patient who suffered from at least one comorbidity from a category was counted in that category. Counted with repetitions. Sorted in descending order of frequency.

Total polypharmacy = intake of at least five drugs (of any kind); Rx polypharmacy = intake of at least five drugs that were prescribed (neglecting OTC)

^{Fi}, Fisher's exact test; MS, multiple sclerosis; N, number of patients; PwP, patients with polypharmacy; Pw/oP, patients without polypharmacy;
p, *p*-value for comparing patients with and without polypharmacy.
