# Supplementary material for: Prevalence and Severity of Potential Drug–Drug Interactions in Patients with Multiple Sclerosis with and without Polypharmacy
Source: Pharmaceutics. 2022 Mar 8;14(3):592. doi: 10.3390/pharmaceutics14030592 (PMC8949310; doi:10.3390/pharmaceutics14030592)
Supplement: Supplementary file 1 [file pharmaceutics-14-00592-s001.zip › Supplement Table S2.pdf]

Supplement Table S2. Prevalence of pDDIs in MS patients stratified by different comorbidity groups.

Comorbidity	Amount of patients with respective comorbidity that have at least one pDDI, N (%)	Amount of patients without respective comorbidity that have at least one pDDI, N (%)	p^{Fi}
**Cardiovascular**	133 (78.2%)	267 (58.4%)	**<0.001**
**Chronic inflammatory**	8 (72.7%)	392 (63.6%)	0.754
**Dermatological**	14 (60.9%)	386 (63.9%)	0.826
**Endocrinological**	34 (69.4%)	366 (63.3%)	0.442
**Ear-nose-throat**	5 (83.3%)	395 (63.6%)	0.426
**Gastrointestinal**	48 (72.7%)	352 (62.7%)	0.136
**Hematological**	5 (71.4%)	395 (63.7%)	1.000
**Metabolic**	76 (68.5%)	324 (62.8%)	0.278
**Neurological**	90 (76.9%)	340 (61.9%)	**0.011**
**Ophthalmological**	19 (82.6%)	381 (63.1%)	0.075
**Orthopaedic**	63 (80.8%)	337 (61.4%)	**0.001**
**Pain**	3 (60.0%)	397 (63.8%)	1.000
**Psychiatric**	103 (85.1%)	297 (58.7%)	**<0.001**
**Pulmonary**	16 (59.3%)	384 (64.0%)	0.683
**Urological/ gynecological**	38 (74.5%)	362 (62.8%)	0.128
**Others**	44 (64.7%)	356 (63.7%)	0.895

Percentages show how many patients with and without at least one comorbidity of each comorbidity group had at least one pDDI (of any severity). Sorted in descending alphabetical order.

^{Fi}, Fisher's exact test; MS, multiple sclerosis; pDDI, potential drug-drug interaction; *p*, *p*-value for comparing patients with and without polypharmacy.
